# Quantitative Phosphoproteomic Analysis Identifies Activation of the RET and IGF-1R/IR Signaling Pathways in Neuroblastoma

**DOI:** 10.1371/journal.pone.0082513

**Published:** 2013-12-11

**Authors:** Bradley D. DeNardo, Michael P. Holloway, Qinqin Ji, Kevin T. Nguyen, Yan Cheng, Marcus B. Valentine, Arthur Salomon, Rachel A. Altura

**Affiliations:** 1 Division of Pediatric Hematology-Oncology, Department of Pediatrics, The Warren Albert School of Medicine at Brown University, Providence, Rhode Island, United States of America; 2 Department of Chemistry, Brown University, Providence, Rhode Island, United States of America; 3 St. Jude Comprehensive Cancer Center Cytogenetic Shared Resource, St. Jude Children’s Research Hospital, Memphis, Tennessee, United States of America; 4 Department of Molecular and Cellular Biochemistry, Brown University, Providence, Rhode Island, United States of America; Rutgers University, United States of America

## Abstract

Neuroblastoma is an embryonal tumor of childhood with a heterogenous clinical presentation that reflects differences in activation of complex biological signaling pathways. Protein phosphorylation is a key component of cellular signal transduction and plays a critical role in processes that control cancer cell growth and survival. We used shotgun LC/MS to compare phosphorylation between a human *MYCN* amplified neuroblastoma cell line (NB10), modeling a resistant tumor, and a human neural precursor cell line (NPC), modeling a normal baseline neural crest cell. 2181 unique phosphorylation sites representing 1171 proteins and 2598 phosphopeptides were found. Protein kinases accounted for 6% of the proteome, with a predominance of tyrosine kinases, supporting their prominent role in oncogenic signaling pathways. Highly abundant receptor tyrosine kinase (RTK) phosphopeptides in the NB10 cell line relative to the NPC cell line included RET, insulin-like growth factor 1 receptor/insulin receptor (IGF-1R/IR), and fibroblast growth factor receptor 1 (FGFR1). Multiple phosphorylated peptides from downstream mediators of the PI3K/AKT/mTOR and RAS pathways were also highly abundant in NB10 relative to NPC. Our analysis highlights the importance of RET, IGF-1R/IR and FGFR1 as RTKs in neuroblastoma and suggests a methodology that can be used to identify potential novel biological therapeutic targets. Furthermore, application of this previously unexploited technology in the clinic opens the possibility of providing a new wide-scale molecular signature to assess disease progression and prognosis.

## Introduction

Neuroblastoma is an embryonal tumor of the sympathetic nervous system that is remarkable for its heterogeneity, including both its biology and clinical behavior [1]. The broad spectrum of neuroblastoma clinical disease encompasses very low-risk infants whose tumors are treated with surgical resection alone and a subset of patients with high-risk factors whose disease treatment involves an intensive multimodal approach including dose-intensive chemotherapy, surgery, stem cell transplant, radiotherapy, and immunotherapy [2]. Despite this aggressive approach to treatment, high-risk neuroblastoma has a 5 year overall survival of only 40% [3]. 

Patients have been traditionally risk-stratified according to tumor-associated biologic factors including *MYCN* gene amplification, DNA ploidy, and loss of heterozygosity (LOH) of chromosomes 1p and 11q [4,5]. In addition, germline mutations resulting in familial neuroblastoma have been identified, including *ALK* and *PHOX2B* [6,7]. A genome-wide analysis currently being performed by the Children’s Oncology Group has revealed several single-nucleotide-polymorphism variations in *FLJ22536* and *BARD1* as being associated with the development of neuroblastoma [8]. Additional genomic approaches have demonstrated *LMO1* as a neuroblastoma oncogene [9]. More recently, gene expression profiling studies have identified a unique 59 gene neuroblastoma tumor signature that is associated with an unfavorable prognosis [10]. A similar approach has revealed a 144 gene panel that is able to accurately risk-stratify patients and predict prognosis [11]. 

Despite these advances in neuroblastoma genomics, our understanding of the complex cell signaling pathways regulating high-risk neuroblastoma growth and metastasis remains limited, and translational gains in clinical outcome remain small [12,13]. These cell signaling cascades are known to be dependent on tyrosine phosphorylation, as multiple tyrosine kinases have been implicated in tumorigenesis [14]. Activating mutations of the *ALK* receptor tyrosine kinase (RTK) have been identified in up to 15% of high-risk neuroblastomas and may occur in both familial and sporadic cases of the disease [6,15]. Activation of ALK results in signaling via the PI3K/Akt, MAPK, and PLCγ pathways leading to cell growth and survival [12]. Constitutive ALK activation demonstrates transforming potential and is associated with high-risk disease [15-17]. As such, several therapeutic options have been developed for ALK-positive neuroblastoma, and the small molecule inhibitor Crizotinib has displayed encouraging results in early phase pediatric trials [18]. TrkB is a RTK that also signals via the PI3K/Akt and MAPK signaling pathways. TrkB is expressed in many *MYCN*-amplified neuroblastoma tumors [19]. TrkB signaling is known to contribute to the aggressive nature of these poor-prognosis tumors and constitutively active TrkB demonstrates malignant transformation potential [20,21]. The non-receptor tyrosine kinases Src and focal adhesion kinase (FAK) have also been implicated in high-risk neuroblastoma biology [22,23]. More recently, the DNA damage response protein (DDR) checkpoint kinase 1 (Chk1) has been identified as being overactive in neuroblastoma. Inhibition of Chk1 and DDR pathway signaling has been efficacious in the treatment of neuroblastoma xenografts [24,25]. Taken together, it is clear that tyrosine kinase activity plays a vital role in driving neuroblastoma oncogenesis. 

Reversible phosphorylation of protein tyrosine, serine, and threonine residues, allows for the propagation of intracellular signaling cascades [14]. The wide-scale analysis of phosphorylation events within a given system is now feasible through the methods of phosphoproteomics [26,27]. Quantitative phosphoproteomics facilitates the relative quantitation of phosphorylation changes between different samples [28]. The aim of this current study was to identify new molecular signatures within the complex signaling networks of a neuroblastoma cell line harboring features of high-risk disease. Shotgun LC/MS was used to compare phosphorylation between a human *MYCN* amplified neuroblastoma cell line (NB10), modeling a resistant tumor, and a human neural precursor cell line (NPC), modeling a normal baseline neural crest cell. In a typical shotgun phosphoproteomics experiment, cell-derived proteins are first digested by trypsin to peptides followed by enrichment of the resulting phosphopeptides using titanium dioxide (TiO) beads or immunoaffinity purification (IAP) with anti-phosphotyrosine resin. The resulting collection of phosphopeptides is sequenced confidently using LC/MS/MS and fold changes for each phosphopeptide are calculated through comparison of selected ion chromatogram peak areas from the NB10 and NPC cell lines. Replicate experiments provide multiple hypothesis corrected statistical assessment of significant alterations in the phosphoproteome of the two types of cells. 

As neuroblastoma is derived from the neural crest lineage, our results provide a model of tumor-specific activation and deactivation signaling events. Phosphopeptides derived from proteins specific to the PI3K/Akt/mTor and the Raf/MEK/ERK pathways were significantly increased in neuroblastoma cells. Additionally, phosphopeptides derived from IGF-1R/IR, Ret, and FGFR, known activators of these downstream pathways, were also significantly elevated. These data support the hypothesis that inhibition of a single protein or signaling pathway may not have a sustained therapeutic effect and highlight the need to concurrently target multiple proteins within these separate pathways to achieve maximal anti-tumor effects.

## Materials and Methods

### Cell lines and culture

The NB5, NB7, NB8, NB10 and NB16 cell lines were kind gifts from St. Jude Children’s Research Hospital [29]. The NPC cells were a kind gift from the laboratory of Dr. Brian Kaspar. NB cells were grown in RPMI 1640 (Invitrogen) supplemented with 10% fetal bovine serum (FBS) and 1% penicillin/streptomycin. NB cells were passaged every 3-5 days via trypsinization. NPC cells were cultured in laminin-coated flasks in DMEM/F12, 10% BIT-9500 (StemCell), 1% N2 supplement (Invitrogen), and 20 μg/ml of EGF and FGF2, as described [30].

### Peptide preparation, fractionation and purification of phosphopeptides

Cells were lysed in buffer (8 M urea, 1 mM sodium orthovanadate, 100 mM ammonium bicarbonate, pH 8.0). Lysates were then cleared at 14,000xg for 15 min at 4°C, and protein concentration was measured by the DC Protein Assay (Bio-Rad, Hercules, CA). Lysates were reduced with 10 mM DTT for 20 min at 60°C, followed by alkylation with 55 mM iodoacetamide for 15 min at room temperature in the dark. Cell lysates were then diluted 4-fold with 20 mM HEPES buffer, pH 8.0 and digested with sequencing grade modified trypsin (Promega, Madison, WI) at 1:100 (w/w) trypsin:protein ratio overnight at room temperature. Tryptic peptides were acidified to pH 2.0 by adding 1/20 volume of 20% trifluoroacetic acid (TFA) for a final concentration of 1% TFA, cleared at 1800xg for 5 minutes at room temperature, and desalted using C18 Sep-Pak plus cartridges (Waters, Milford, MA), as described [31], with the exception that TFA was used instead of acetic acid at the same required concentrations. Eluents containing peptides were lyophilized for 48 hours to dryness. 

Phosphopeptides were enriched through two ways. First, phosphotyrosine was enriched through peptide immunoprecipitation by using p-Tyr-100 phosphotyrosine antibody beads (Cell Signaling Technology) as described [32]. 10^8^ cells were used for each biological replicate for this protocol. Protein concentrations were equivalent for each cell line at 3.5 ng/cell. Dry peptides from each cell line were reconstituted in ice-cold immunoaffinity purification (IAP) buffer (50 mM MOPS pH 7.2, 10 mM sodium phosphate, 50 mM NaCl) and further dissolved through gentle shaking for 30 minutes at room temperature and brief sonication in a sonicator water bath. Prior to peptide immunoprecipitation, a 10 pmol portion of synthetic phosphopeptide LIEDAEpYTAK was added to each cell line as an exogenous quantitation standard. Peptide solutions were then cleared at 1800xg for 5 minutes at room temperature, combined with pre-conjugated p-Tyr-100 phophotyrosine antibody beads, and incubated for 2 hours at 4°C. Beads were washed 3 times with IAP buffer and twice with cold Milli-Q water, and eluted with 0.15% TFA. Samples were then desalted using Zip-Tip C18 columns (EMD Millipore, Billerica, MA). 

In a parallel set of analyses, total phosphopeptides were enriched with Titan-sphere Phos-TiO reagents as described [33]. The TiO method is necessary to ascertain changes in the phosphoproteome of peptides containing serine or threonine phosphorylation sites. While this method also provides some information about tyrosine phosphorylated peptides, the relative scarcity of phosphotyrosine in the phosphoproteome requires the combination of phosphotyrosine peptide enrichment by immunoaffinity precipitation (described above) with the TiO protocol to provide the most extensive deep sequencing of the phosphoproteome. 10^7^ cells were used for each biological replicate for this protocol. Dry peptides from each cell line were reconstituted in 0.1% Formic acid, 30% acetonitrile solution. A 500 fmol portion of synthetic phosphopeptide FQpSEEQQQTEDELQDK was added to each cell line as an exogenous quantitation standard. Adsorption of peptides proceeded according to the manufacturer’s protocol. Phosphopeptides were eluted from the Phos-TiO tips by first applying 1% ammonium hydroxide in water. The tips were then treated with 1% ammonium hydroxide in 40% acetonitrile. All samples were dried in a SpeedVac concentrator and stored at -80°C. 

### Automated LC/MS analysis

Three biological replicates were performed for the IAP analysis and 5 biological replicates were performed for the TiO analysis. Each of these samples was analyzed by a fully automated phosphoproteomic technology platform that incorporates peptide desalting and separation via reverse phase chromatography followed by tandem mass spectrometry with static peak parking [34,35]. Briefly, phosphopeptides were eluted into a Linear Trap Quadropole (LTQ) Oribitrap Velos mass spectrometer (Thermo Fisher Scientific) through a PicoFrit analytical column (360 μm outer diameter, 75 μm inner diameter; fused silica with 12 cm of 3μm Monitor C18 particles; New Objective, Woburn, MA) with a reversed-phase (0–70% 0.1 M acetic acid in acetonitrile in 90 minutes). Spectra were collected in positive ion mode and in cycles of one full MS scan in the Oribitrap (m/z 400 to 1800) followed by data-dependent tandem mass spectra (MS/MS) scans in the LTQ Velos, sequentially of the ten most abundant ions in each MS scan with charge state screening for +1, +2, +3 ions and dynamic exclusion time of 30 seconds. The maximum ion time was 100 milliseconds for the LTQ scan and 500 milliseconds for the Orbitrap full scan. Orbitrap resolution was set at 60,000 at precursor level and a normal scan rate was used for fragment level.

### Database analysis

MS/MS spectra were searched against the non-redundant human UniProt Complete Proteome set database containing 72,078 forward and an equal number of reversed decoy protein entries using the Mascot algorithm provided with Matrix Science [36]. Peak lists were generated using Bioworks 3.3 (extract_msn.exe 07/12/07) using a mass range of 600-4500. The Mascot database search was performed with the following parameters: trypsin enzyme cleavage specificity, 2 possible missed cleavages, 7 ppm mass tolerance for precursor ions, 0.5 Da mass tolerance for fragment ions. Search parameters specified a differential modification of phosphorylation (+79.9663 Da) on serine, threonine, and tyrosine residues, a dynamic modification of methionine oxidation (+15.9949 Da), and a static modification of carbamidomethylation (+57.0215 Da) on cysteine. To provide high confidence phosphopeptide sequence assignments, data was filtered for Mowse score (>20 for all charge states) for Mascot results. In addition, a logistic spectral score [34] filter was applied to achieve a final decoy database estimated false discovery rate (FDR) of 1% after final assembly of non-redundant data into heatmaps [37]. To validate the positions of the phosphorylation sites, the Ascore algorithm [38] was applied to all data, and the reported phosphorylation site position reflected the top Ascore prediction. Ascore probabilities are reported in the full data tables for both TiO and IAP analyses (Datasets S1 and S2).

### Quantitation of relative phosphopeptide abundance

 Relative quantification of peptide abundance was performed via calculation of selected ion chromatogram (SIC) peak areas normalized to the SIC peak area of the copurified synthetic exogenous peptide LIEDAEpYTAK or FQpSEEQQQTEDELQDK. Peak areas were calculated by inspection of SICs using in-house software programmed in Microsoft Visual Basic 6.0 based on Xcalibur Development Kit 2.2 (Thermo Fisher Scientific). Quantitative data was calculated automatically for every assigned peptide using the ICIS algorithm available in the Xcalibur Development Kit. A minimum SIC peak area equivalent to the typical spectral noise level of 500 was required of all data reported for label free quantitation.

 A label-free heatmap was generated for quantitative comparison of phosphopeptides in NB10 and NPC cells, as previously described [34]. Any changes (either an increase or decrease of peptide abundance above the average) greater than 100 fold were displayed as the same color as the 100-fold change. Black represents average abundance of a given phosphopeptide, while yellow (blue) represents levels of phosphorylation above (below) the average. Blank heatmap squares indicate data points without a clearly defined SIC peak in any of the replicate analyses. The coefficient of variation (CV) for each heatmap square amongst the replicate data was calculated (Datasets S1 and S2) and represented as a color bar beneath each heatmap square. According to the CV color key, black represents 0% CV and more orange represents a larger CV. Label free *p* values for each phosphopeptide were calculated from the replicate data for NPC cell line compared to NB10 cell line based on *t* statistics for small data set (Methods S1). A *Q* value is defined as the measure of the minimum FDR at which a test can be called significant [39]. For each phosphopeptide, *Q* values for multiple hypothesis tests were calculated based on the determined *p* values using the R package QVALUE, as previously described [40,41]. A white dot on a label free heatmap square indicates that a significant difference (Q value < 5%) was detected between NPC cell line and NB10 cell line for that phosphopeptide (Datasets S1 and S2). A minimum of 3 replicates for each detected phosphopeptide was required for *p* value and *Q* value calculation. To allow for public availability, quantitative phosphoproteomic data was uploaded to the Salomon Research Group laboratory website and can be accessed at http://cellpathway.com/.

### RNA FISH

Cells were cultured on slides in RNase-free PBS, rinsed then permeabilized in PBS, 0.5% v/v Triton X-100 on ice for 5-7 min. Cells were fixed in freshly made, filtered 3% PFA for 10 min at RT then washed twice in 70% v/v ethanol for 5 min each. Slides were stored in 70% ethanol at -20C. Prior to FISH, cells were dehydrated in 80%, 95%, 100% v/v ethanol for 3 min each then air-dried. Cells were hybridized overnight at 37°C (ThermoBrite) with fluorescent probes then washed in freshly made 50% formamide, 2XSSC for 5 min at 42°C, followed by 2XSSC for 5 min each at 42°C. Cells were stained with mounting medium (Vectashield antifade in 0.1µg/ml Dapi), coverslipped and imaged using a confocal microscope.

### Antibodies and inhibitors

The following primary antibodies were used for immunoblotting: β-actin, GSK3β, IGF-1Rα, Insulin receptor α, PI3K p85β, and Ret (Santa Cruz Biotechnology, CA, USA). Secondary antibodies were goat anti-mouse IgG HRP and goat anti-rabbit IgG HRP (Santa Cruz Biotechnology, CA, USA). The Ret inhibitor Vandetanib (ZD6474, Selleck Chemistry, TX, USA) was dissolved in DMSO (Sigma Aldrich, MO, USA) and diluted in RPMI 1640 cell culture medium immediately prior to use. The IGF-1R/IR inhibitor BMS 754807 (Active Biochem, NJ, USA) was also dissolved in DMSO and diluted in RPMI 1640 cell culture medium immediately prior to use.

### siRNA Transfection

NB10 cells were plated in 6-well plates at a seeding density of 150,000-250,000 cells/well and then transfected with IGF-1R siRNA as well as non-targeting siRNA used as a control (Dharmacon). Transfection was performed 48 hours after seeding once cell growth achieved 60-80% confluence. Transfection was performed to final concentrations of 10-40 nM using Lipofectamine RNAiMAX Transfection Reagent (Invitrogen, CA, USA) per the manufacturer’s instructions. Appropriate siRNA knockdown was determined by western blotting.

### Immunoblotting

Cell lysates were prepared in RIPA buffer (50 mM Tris HCl, pH 7.5, 150 mM NaCl, 1% NP-40, 0.1% SDS, 1% NaDeoxycholate) and supplemented with Protease Inhibitor Cocktail (Sigma Aldrich, MO, USA) as well as a phosphatase inhibitor (Calbiochem, EMD Millipore, Germany). Protein concentration was normalized using a bicinchoninic acid (BCA) protein assay (Pierce, IL, USA). Cell lysates were separated by sodium dodecyl sulfate-polyacrylamide gel electrophoresis (SDS-PAGE) then transferred to hydrophobic polyvinylidenedifluoride (PVDF) membrane (BIO-Rad, CA, USA). Immunoblotting with the specific antibodies was then performed followed by chemoiluminescent detection using the Amersham ECL Prime Western Blotting Detection Reagent (GE Healthcare, Sweden). Immunoblotting was performed a minimum of 3 times.

### Cell viability assays

NPC and NB cells were seeded in 96-well plates at a density of 5,000-10,000 cells/well. When cell growth reached 50-75% confluence, cells were grown for 24-72 hours in the presence of the respective inhibitor. Cell viability was measured using the CellTiter 96 AQueous One Solution Cell Proliferation Assay (Promega, WI, USA), according to the manufacturer’s instructions.

## Results

### Phosphoproteomic profiling of neuroblastoma and neural progenitor cells

To determine the differences in phosphopeptide abundance in neuroblastoma cells compared with normal neural crest-derived cells, we isolated proteins from the NB10 cell line, representing a high-risk *MYCN* amplified cell line, and from an NPC line, representing a control human neural crest cell line. Phosphopeptides were prepared and analyzed in three to five replicate experiments using two different protocols, as detailed in the Methods section. For the total phosphoproteome, phosphopeptides were isolated using titanium dioxide purification. For highly sensitive detection of the tyrosine-phosphoproteome, phosphopeptides were isolated using immune-affinity precipitation (IAP) with a phosphotyrosine antibody. Quantitation was achieved using normalization of label free peak areas to a standard exogenous phosphopeptide added to cell lysates prior to IAP. 

From the total phosphoproteome analysis, we identified 2598 unique serine, threonine, and tyrosine phosphopeptide sequences in both NB10 and NPC cell lines using a 1% false discovery rate (FDR). Amongst these peptide sequences, 2181 unique phosphorylation sites were identified residing on 1171 proteins (Table S1). Serine phosphorylation represented 1768 sites which was 81% of all phosphorylation events, followed in abundance by threonine phosphorylation (239 sites, 10%), and tyrosine phosphorylation (174 sites, 8%). A total of 176 phosphorylation sites residing on 151 protein kinases were also observed (Table S2). A significant proportion of phosphorylation sites identified here had not previously been reported in neuroblastoma cells, highlighting the strength of this experimental approach. Among the 176 sites identified on protein kinases, for example, 111 had not been previously identified in neuroblastoma. 

Our experimental approach also allowed for a relative quantitation of phosphopeptides. Of the 2598 unique phosphopeptide sequences, 2322 were significantly increased (Q value <5%) in the NB10 cells relative to the NPC cells and 277 were increased in the NPC cells (Table S1). A subset of tyrosine phosphopeptides with the largest increase in abundance in NB10 relative to NPC from the IAP analysis is shown in Table S3.

### Gene ontology and pathway analysis

**Figure 1:**
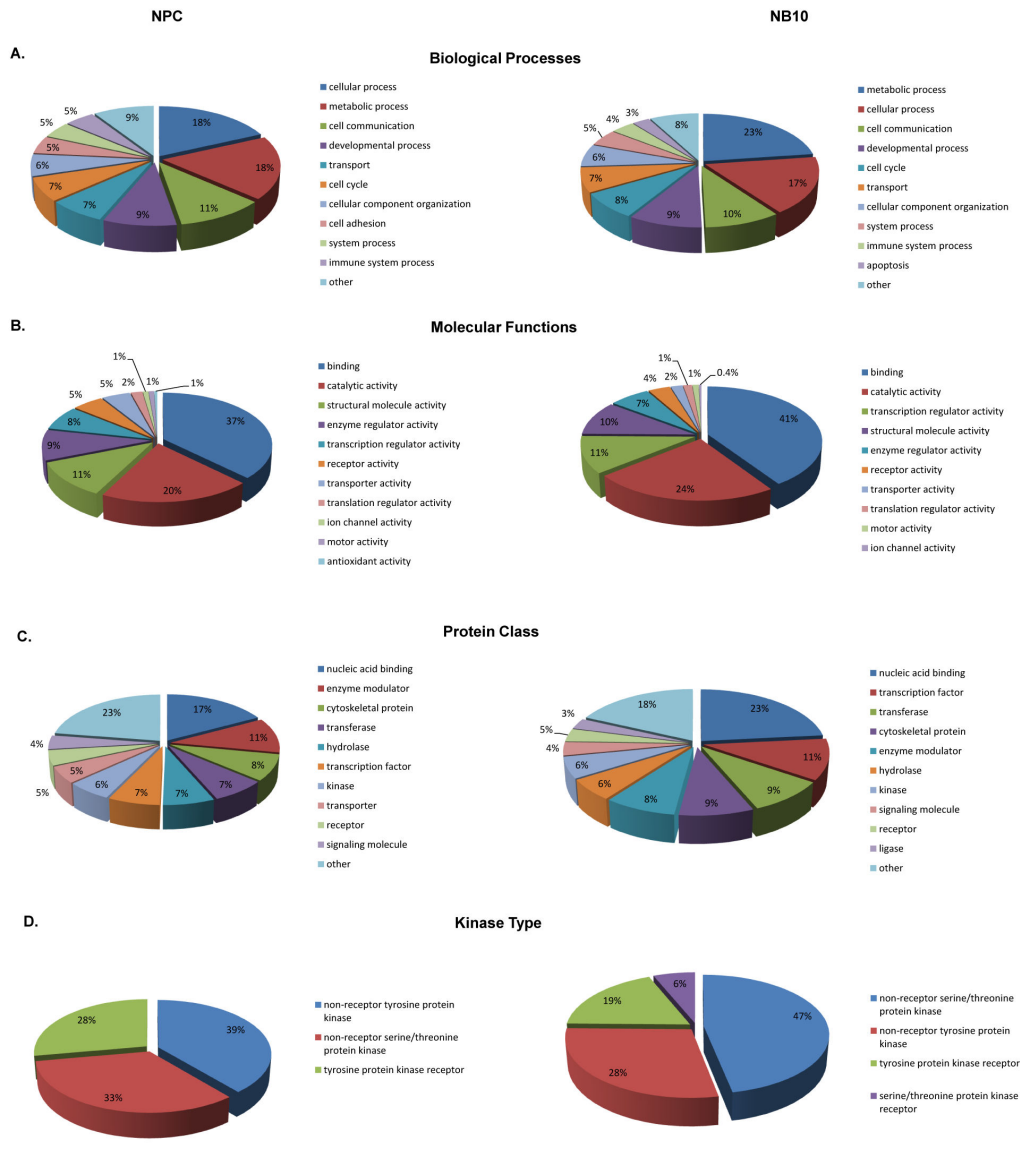
Gene ontology distribution of phosphopeptides in the NPC and NB10 phosphoproteome. Distributions of the human neural precursor (NPC) cell line phosphoproteome are displayed on the left for each analysis, while distributions of the human MYCN amplified neuroblastoma cell line (NB10) phosphoproteome are displayed on the right. Each category is represented by the percentage of the total number of genes included in that analysis. The most abundant ten categories for each analysis are presented when applicable.

To gain an understanding of the type of biological processes as well as molecular functions associated with the phosphopeptides identified in this study, we made use of the Gene Ontology (GO) metadata in the Human Protein Reference Database [42]. Categorizing phosphopeptides by biological processes showed that the highest percentages accounted for in both NB10 and NPC cells were metabolic (23% NB10, 18% NPC) and cellular processes (17% NB10, 18% NPC) (Figure 1A). Categorizing phosphopeptides by molecular functions showed that protein binding events were dominant in both NB10 and NPC (41% NB10 and 37% NPC), while catalytic activity was also highly prevalent (24% NB10, 20% NPC) (Figure 1B).

To categorize the types of proteins and kinases associated with the phosphopeptides observed in this study, we used the PANTHER (Protein ANalysis THrough Evolutionary Relationships) classification system [43,44]. This analysis showed that nucleic acid binding was the most abundant protein class represented in both NB10 (23%) and NPC (17%) cells (Figure 1C). The protein subtypes represented by the remaining phosphopeptides diverged. Transcription factors (11%) and transferase proteins (9%) represented the next most abundant categories in NB10 cells, while enzyme modulators (11%) and cytoskeletal proteins (8%) were next most abundant in NPC cells. A subset analysis of kinase proteins representing the phosphopeptides detected showed that non-receptor serine/threonine protein kinases were highly represented in NB10 cells (47%), while non-receptor tyrosine protein kinases were most prevalent in NPC cells (39%) (Figure 1D). Abundant kinases observed in this analysis are represented in Table S2.

**Figure 2:**
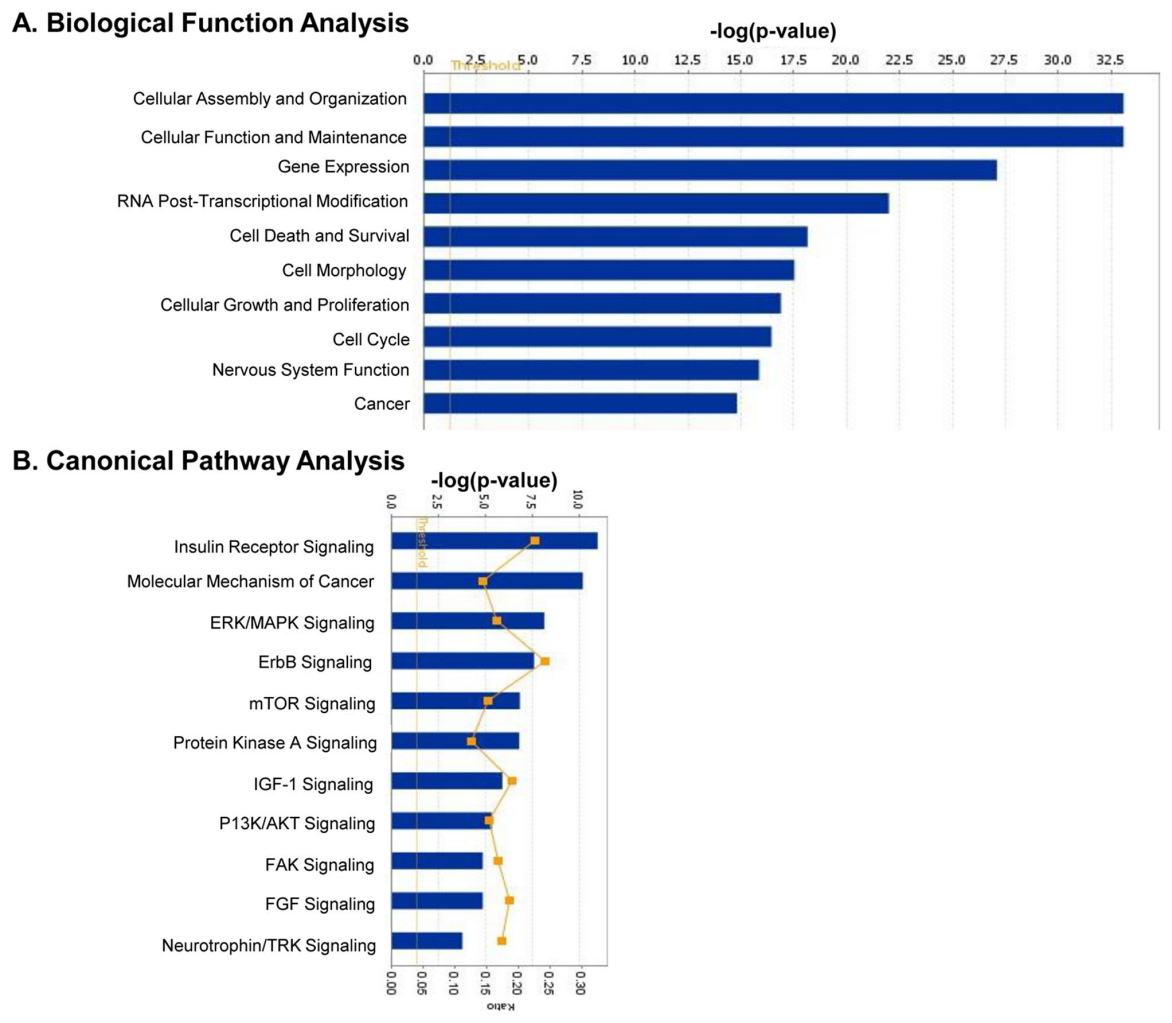
Biological function and canonical pathway analysis of signaling pathways regulated by identified phosphopeptides in NB10 cells. (A) Ingenuity Systems Pathway Analysis (IPA) software was used to determine the highest biologically active functions in the NB10 cell phosphoproteome. (B) IPA software was also used to determine canonical pathways highly represented in the NB10 phosphoproteome. Phosphopeptides found to be relatively overexpressed in the NB10 cell line compared to the NPC cell line were analyzed.

To understand the potential biological signaling pathways regulated by the identified phosphopeptides in the neuroblastoma cells, we used Ingenuity Systems Interactive Pathway Analysis software (IPA) (Figure 2). Biological functions highly represented by the phosphopeptides detected in the NB10 cell line included those regulating cellular assembly/function and gene expression (Figure 2A). Cell death/survival, growth, and nervous system development were also highly represented in the NB10 cells. Biological functions known to be associated with cancer development were similarly represented in the NB10 cells. Specifically, cellular transformation mechanisms were highly represented in the NB10 cell line compared to the NPC cell line by this analysis. Canonical pathway analysis showed that insulin receptor signaling was the most statistically significant elevated pathway in the NB10 cell line (Figure 2B). Similarly, the molecular mechanisms of cancer canonical pathways were highly represented in the NB10 cells. Other such pathways that were highly represented in the NB10 cells included the ERK/MAPK and mTor signaling pathways, which are known to play a role in neuroblastoma tumorigenesis [45-48]. 

### Raf/MEK/ERK and PI3K/Akt/mTor signaling pathways are induced in neuroblastoma

**Figure 3:**
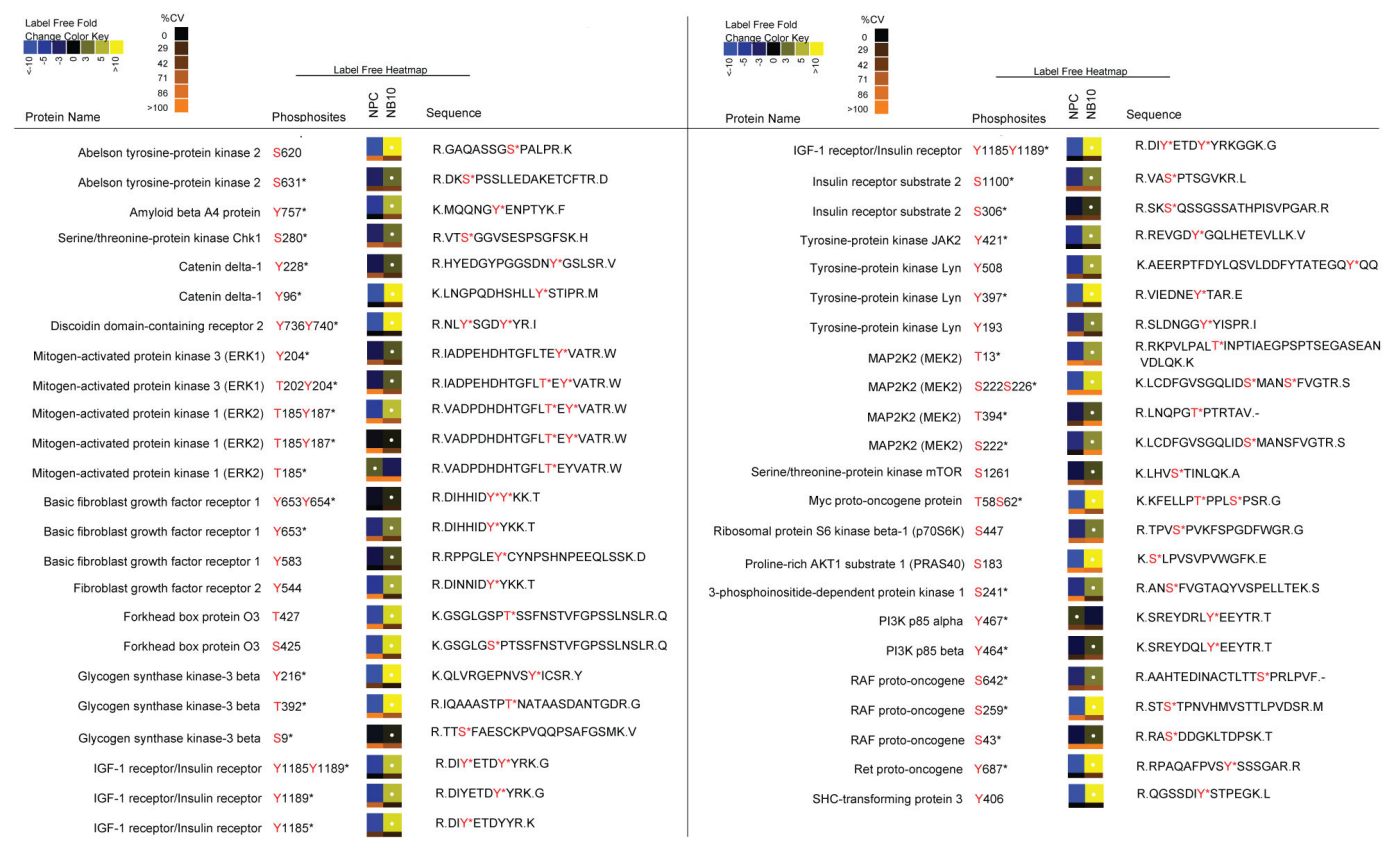
Relative quantitative expression of selected phosphopeptides in the NB10 and NPC cell lines. Changes in phosphorylation abundance are represented as heatmaps from 5 replicate experiments as described in the Methods. These label free heatmaps represent the change of abundance of phosphopeptides in control NPC cells relative to NB10 neuroblastoma cells. In these heatmaps, black represents average phosphopeptide abundance for that particular peptide between NPC and NB10 cells, while yellow (blue) represents phosphopeptide levels above (below) the average. White dots on heapmaps represent peptides with a false discovery rate less than 1% for significant changes in phosphopeptide abundance compared with the minimal average abundance. The coefficient of variation (CV) for each heatmap square amongst the 5 replicate experiments was calculated and represented as a color bar on the bottom of that heatmap. According to the CV color key, black represents 0% CV and more orange represents larger CV. According to Human Protein Reference Database (HPRD) Release 7, phosphorylation sites previously discussed in the literature are marked with * if identified using traditional approaches such as site-directed mutagenesis.

From the total phosphoproteome and IAP analyses, we observed many phosphopeptides on proteins in the NB10 cell line that are implicated in tumorigenesis. These phosphopeptides were statistically increased in the NB10 cell line relative to the NPC cell line (Q value <0.05, Figure 3). Included in this group of peptides were several downstream mediators of the PI3K/Akt/mTor pathway, a known oncogenic signaling pathway in human cancer [49-51]. One such example is the p85β subunit of PI3K, phosphorylated at Y464 that was significantly increased in NB10. PI3K is a heterodimeric enzyme consisting of the catalytic subunit, p110, and its regulatory subunit p85, of which there are two major isoforms, p85α and p85β. Phosphorylation of these p85 isoforms relieves the inhibitory function of this subunit and activates PI3K [52]. The isoform p85α was also identified in this analysis, demonstrating overabundance of this phosphopeptide in NPC. Multiple phosphopeptides from downstream mediators of PI3K were highly abundant in NB10 as well, including PDK1, GSK3β, FOXO3 and mTor, suggesting overall induction of this pathway. Phosphorylation of Akt and PTEN, a phosphatase antagonist of PI3K, were not identified in this analysis. An abundance of a phosphopeptide containing S241 was observed in NB10 on PDK1, a known activating phosphorylation site [53,54]. A peptide containing phosphorylation at S425 of FOXO3, a tumor suppressor protein, was more abundant in NB10. This post-translational modification causes inhibition of FOXO3 tumor suppressor activity [55]. A phosphopeptide containing S9 and another containing Y216 on GSK3β, a ubiquitously expressed serine/threonine kinase implicated in tumorigenesis, was more abundant in NB10 [56]. 

**Figure 4:**
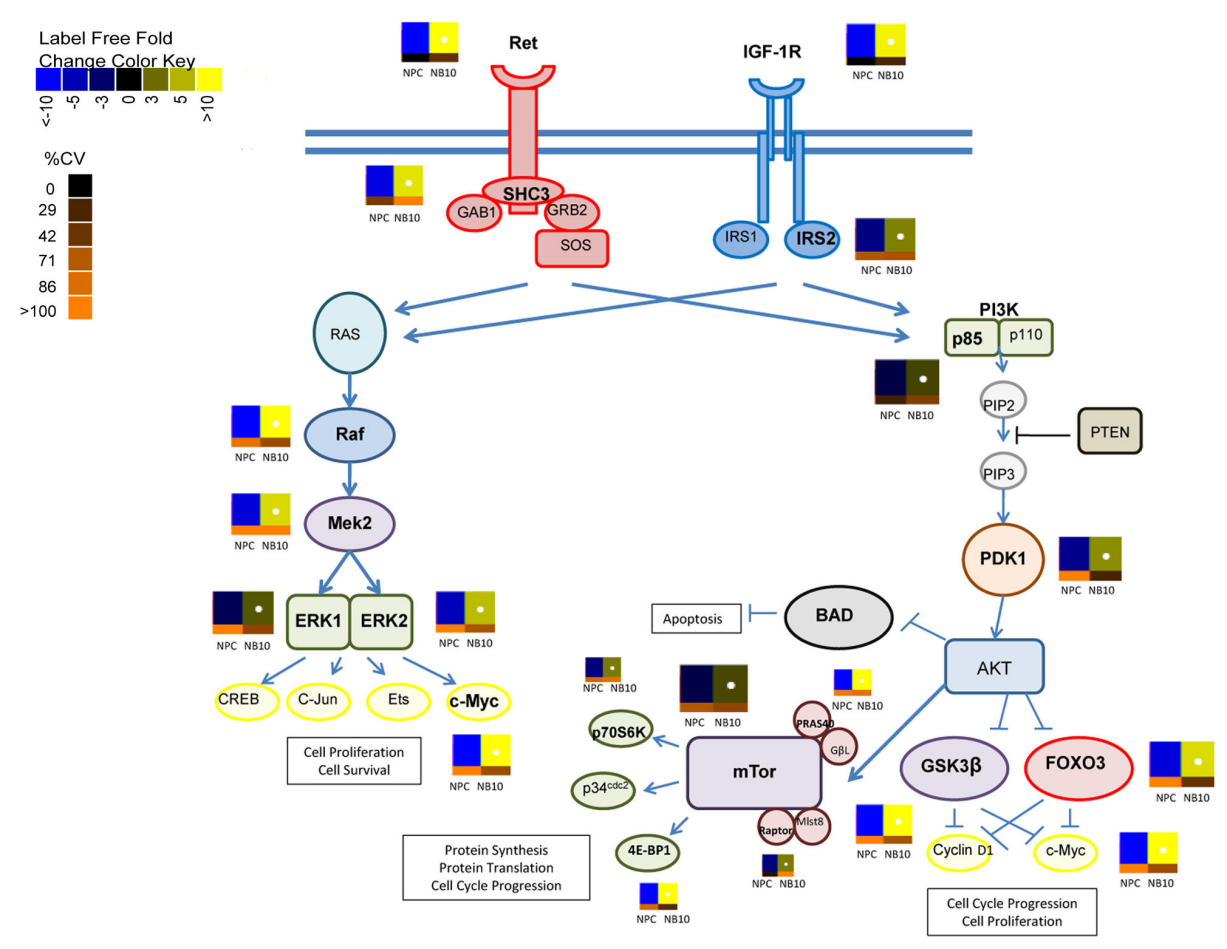
Global activation of Ret and IGF-1R/IR pathways in neuroblastoma. Shown are the Ret and IGF-1R/IR signaling pathways and their downstream mediators. Identified phosphopeptides in the NB10 cell line compared with the NPC cell line are indicated by adjacent heatmaps that demonstrate the degree of increase or decrease in the abundance of these phosphopeptides.

mTor is a serine/threonine kinase with a central role in regulating cell proliferation [57-59]. A significantly increased phosphopeptide containing S1261 from mTor, which acts to promote mTORC1 activity, was identified in NB10 (Figures 3 and 4). Correspondingly, phosphopeptides from the mTor partner proteins, Raptor and PRAS40 were more highly abundant in NB10. The transcriptional regulators p70S6K and 4E-BP1 are two major targets of mTor phosphorylation [60]. Phosphopeptides from both were more highly abundant in NB10, which may indicate upregulation of mTor activity in these neuroblastoma cells.

Phosphopeptides derived from downstream mediators of the Raf/MEK/ERK signaling pathway were more abundant in the NB10 cell line (Figures 3 and 4). Raf, MEK2, ERK1, and ERK2 phosphopeptides were elevated in NB10 relative to NPC, which may indicate upregulation of this signaling cascade. Ras was not identified in this analysis. Raf was phosphorylated at S43 and S259, two known inhibitory phosphorylation sites [61,62]. Most of the phosphorylation sites on MEK2 (S222, S226, T13, T394), ERK1 (T202, Y204), and ERK2 (T185, Y187) identified in this analysis activate these kinases [56,63]. Phosphopeptides from the transcription factor c-Myc, an end-target of both PI3K/Akt/mTor and Raf/MEK/ERK signaling were elevated in the neuroblastoma cell line as well (Figures 3 and 4). These results are consistent with the global pathway analysis performed using IPA software and highlight the potential dual activation of both the PI3K/Akt/mTor pathway and the Raf/MEK/ERK pathway in the neuroblastoma cells.

### Multiple receptor tyrosine kinases (RTKs) are activated in neuroblastoma

Multiple phosphorylated RTKs were identified in the NB10 cell line. IGF-1R/IR is an RTK that is known to play an essential role in embryonal tumor cell biology [64,65]. Upon activation, IGF-1R/IR initiates intracellular signaling via the PI3K/Akt/mTor and Raf/MEK/ERK pathways. Our analysis found significantly elevated phosphopeptides from IGF-1R/IR in the NB10 cell line (Figure 3). Phosphorylation of IGF-1R/IR was observed at Y1185 and Y1189 in this analysis. These phosphorylation sites have been previously described to result in activation of insulin receptor (IR) specifically [66], but are not described in IGF-1R. IGF-1R and IR are homologous structures that spontaneously form heterodimers [65,67]. They could not be distinguished by this analysis because the peptide sequences in these regions are identical in IGF-1R and IR. Major substrates of IGF-1R/IR signaling were also identified in this analysis, including IRS-2 and Shc3. An elevated level of phosphopeptides from NB10 on IRS-2 and Shc3 were identified in this analysis, while IRS-1, an additional IGF-1R/IR substrate, was not identified (Figure 3). Neuroblastoma cells are known to exclusively express IRS-2 over IRS-1 [68], consistent with these results.

We also identified a phosphopeptide from the proto-oncogene Ret with high abundance in the NB10 cell line compared with the NPC cell line (Figure 3). This RTK is highly expressed on neurons and neural crest-derived cells and initiates signaling via multiple signal transduction pathways, including PI3K/Akt/mTor and Raf/MEK/ERK [69]. Such activation results in cell proliferation, migration, differentiation, and survival. Ret phosphorylation was observed at Y687, a site that is known to directly interact with SHP2 [70], a protein tyrosine phosphatase that activates PI3K/Akt/mTor and Raf/MEK/ERK pathways. In addition, a phosphopeptide from Shc3, a known substrate of Ret [71,72], was elevated in NB10 relative to NPC. Taken together, this analysis implicates activation of IGF-1R/IR and Ret as drivers of intracellular signaling via upregulation of the PI3K/Akt/mTor and Raf/MEK/ERK pathways.

Additional phosphopeptides from RTK’s with increased abundance in the NB10 cell line relative to the NPC cell line included FGFR1 and FGFR2 (Figure 3). Similarly, other important phosphopeptides from non-receptor tyrosine kinases were identified in both cell lines with high abundance in NB10 relative to NPC. These phosphopeptides included JAK2, ABL2, and Lyn as well as CDK2 and CDK5, non-receptor serine/threonine kinases that act as cell cycle regulators. A phosphopeptide derived from BAD, a Bcl-2 family member that normally acts to promote cell death [73], was identified in its inactivated form, with phosphorylation at S118 in the NB10 cell line alone. 

### Immunoblot and RNA FISH confirm activation of IGF-1R/IR and Ret

**Figure 5:**
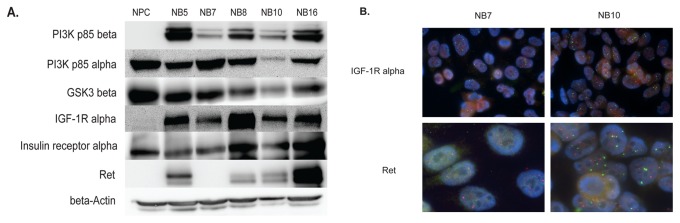
Western blot and RNA fluorescence in situ hybridization (FISH) analysis. (A) Protein lysates from the indicated cell lines were separated by SDS-PAGE and immunoblotted with the indicated antibodies. (B) Cells were grown on slides then fixed, permeabilized and RNA probed for Ret and IGF-1Rα.

To examine the total protein levels corresponding to representative phosphopeptides identified by LC/MS analysis, we performed western blots using total cell lysates isolated from *MYCN* amplified (NB7, NB8, NB10) and *MYCN* non-amplified cell lines (NB5, NB16) (Figure 5A). High levels of the p85β subunit of PI3K were observed in all neuroblastoma cell lines tested with low levels in NPC. Conversely, the p85α subunit showed high levels in NPC cells with low levels in NB10. These results could suggest that the high level of phosphopeptide from the p85β subunit of PI3K that was identified in the NB10 cells is generated from high amounts of total protein. Western blot for total GSK3β protein demonstrated expression in all cell lines examined, including higher expression in NPC with lower expression in NB10. By contrast, the phosphoproteomic analysis demonstrated higher levels of phosphopeptide from GSK3β in NB10 compared to NPC. These findings could suggest that the phosphorylated form of GSK3β is induced in the NB10 cells. IGF-1Rα was expressed in all 5 neuroblastoma cell lines, but was absent in NPC cells, correlating with the phosphoproteomic results. IRα was expressed in all examined neuroblastoma cell lines as well as in the NPC cell line, suggesting in combination with the proteomic data that the stoichiometry of phosphorylation of IRα may be specifically increased in NB10. Ret was expressed in the NB5, NB8, NB10, and NB16 cell lines, but was absent in the *MYCN*-amplified NB7 cell line and the NPC cell line, correlating with the phosphoproteome results. A limitation of this type of analysis is that immunoblots represent total protein levels and not phosphorylation changes between proteins. Anti-phospho specific antibodies were unavailable for the majority of the phosphopeptides that we identified in the phosphoproteome analysis. 

We used quantitative RNA FISH to further examine the RNA levels of Ret and IGF-1Rα in two neuroblastoma cell lines (Figure 5B). FISH for IGF-1Rα showed high levels of RNA expression in both NB7 and NB10 cell lines. FISH for Ret showed low levels of RNA in NB7 and high levels in NB10. These results parallel the results of the immunoblot analysis. 

### Inhibition of Ret and IGF-1R/IR result in decreased tumor cell viability

**Figure 6:**
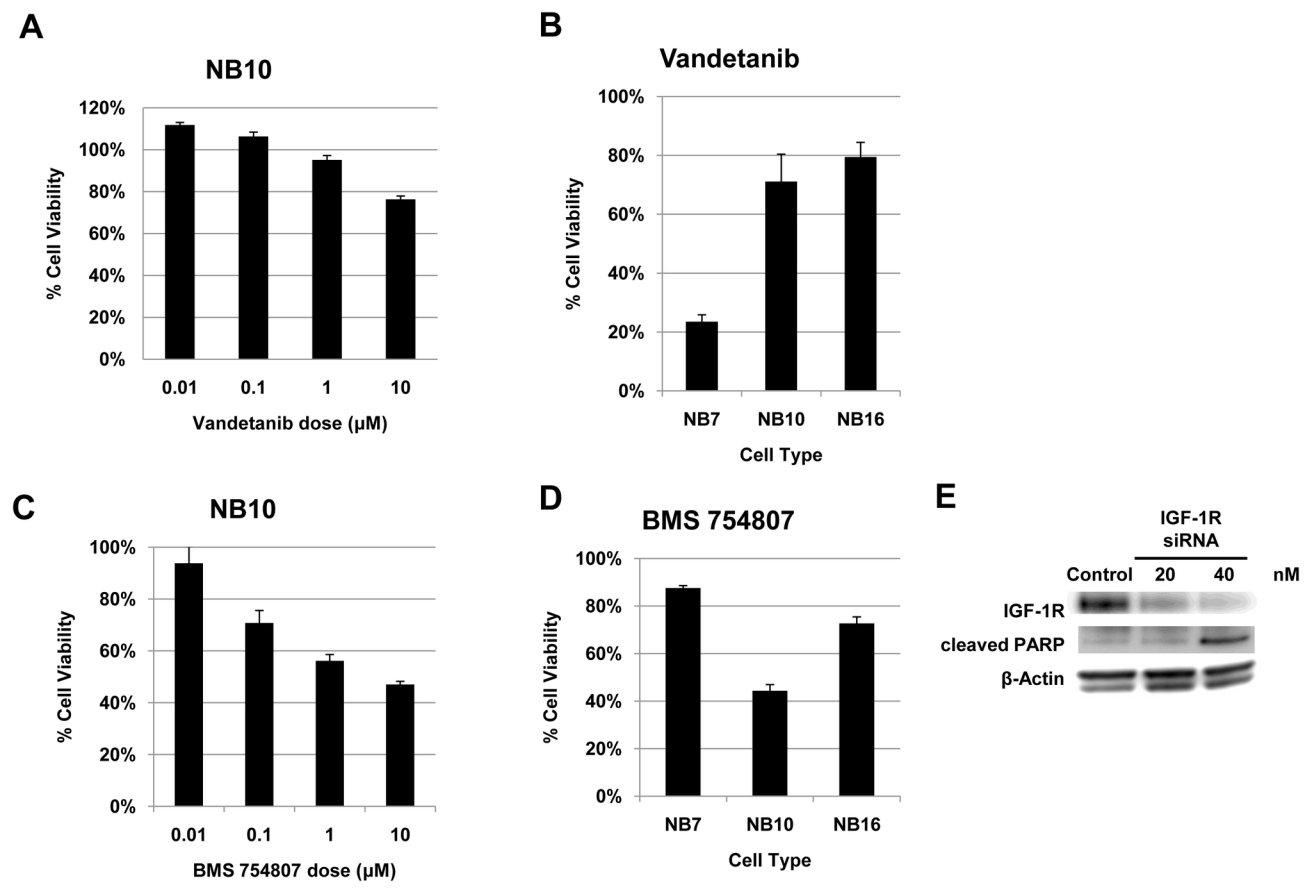
Inhibition of Ret and IGF-1R decreases neuroblastoma tumor cell viability. (A) NB10 cells were treated with varying doses of Vandetanib and cell viability was assessed after 72 hours. (B) NB7, NB10, and NB16 cell lines were treated with 10 μM Vandetanib and cell viability was assessed at 48 hours. (C) NB10 cells were treated with varying doses of BMS 754807 and cell viability was assessed at 72 hours. (D) NB7, NB10, and NB16 cell lines were treated with 10 μM BMS 754807 and cell viability was assessed at 48 hours. (E) NB10 cells were transfected with IGF-1R siRNA and control siRNA. Protein lysates were separated by SDS-PAGE and immunoblotted with the indicated antibodies.

To test the effects of inhibiting Ret signaling on tumor cell viability, we treated 3 different cell lines (NB7, NB10, NB16) with the multi-kinase inhibitor Vandetanib (ZD6474), currently in human trials for other cancers [74,75], and measured cell viability using an MTS-based assay. Vandetanib acts by inhibiting several tyrosine kinases including Ret, VEGFR2, EGFR, BRK, TIE2, and members of the EPH receptor kinase and Src kinase families [76,77]. Treatment of NB10 cells with Vandetanib resulted in a decrease in cell viability at doses of 1 to 10μM (Figure 6A), in keeping with prior reports [78,79]. Treatment of additional NB cell lines with Vandetanib demonstrated a similar effect, with the most potent growth inhibitory effect noted in NB7 cells (Figure 6B). These effects could be mediated, in part, through inhibition of Ret activation of cell survival pathways (PI3K/Akt/mTor and Raf/MEK/ERK). However, the inhibitory effects of Vandetanib on VEGFR2 and EGFR signaling cannot be distinguished with respect to neuroblastoma cell survival. A small molecule inhibitor specific to Ret alone is not yet available. 

To test the effects of inhibiting IGF-IR activation on tumor cell viability, we treated NB7, NB10, and NB16 cells with BMS 754807, a small molecule inhibitor of the insulin-like growth factor 1 receptor/insulin receptor family kinases [80,81]. BMS 754807 is an orally available, reversible inhibitor of IGF-1R and IR that has undergone preclinical testing in breast and other solid tumor models [80,82]. NB10 cells treated with BMS 754807 demonstrated decreased cell viability in a dose-dependent manner (Figure 6C). BMS 754807 treatment also resulted in a decrease in cell viability in additional NB cell lines at similar time points (Figure 6D). Knockdown of IGF-1R in NB10 cells correlated with an increase in protein levels of the pro-apoptotic marker cleaved PARP (Figure 6E), suggesting that the observed decrease in cell viability is associated with an increase in cell death via this pathway.

## Discussion

Intracellular signaling networks are regulated in part by the reversible phosphorylation of serine, threonine, and tyrosine residues. As such, protein kinases and phosphatases play an integral role in regulating nearly all cellular processes, including cell growth, differentiation, migration, and apoptosis [83]. Dysregulation of these signaling networks is implicated in the pathogenesis of many malignancies, including neuroblastoma [84,85]. Tyrosine phosphorylation in particular is known to have a prominent role in cancer, with nearly half of the 90 known tyrosine kinases implicated in cancer, despite the fact that tyrosine phosphorylation itself accounts for less than 1% of the total phosphoproteome [86]. Perturbation of protein tyrosine kinase signaling results in malignant transformation, as seen in ErbB2/HER2/Neu overexpression in breast cancer and the NPM-ALK fusion gene in anaplastic large cell lymphoma [87,88]. Many human tumors have aberrant tyrosine kinase activity resulting from gain-of-function (GOF) mutations, for example GOF mutations in the proto-oncogene RAS results in colorectal carcinoma [14,89]. Given the contribution of protein tyrosine kinase activity to malignant pathogenesis, pharmacologic targeting of these kinases has been fervently pursued over the past 20 years. This has resulted in excellent treatment responses, such as with Imatinib and other similar inhibitors for CML, Trastuzumab in breast cancer, Cetuximab in colorectal cancer, and Erlotinib for non-small cell lung cancer [90]. 

The role of protein phosphorylation in complex intracellular signaling cascades has previously been studied at the level of individual proteins. Current systems biology approaches utilizing tandem mass spectrometry and shotgun proteomics now allow for the global analysis of all phosphorylated proteins within a given system in the setting of a single experiment [91]. Quantitative phosphoproteomics provides an analysis of the specific phosphorylated peptides of the individual proteins within an entire proteome [92]. By quantifying these signaling networks within a cancer cell, one is better able to correlate this data with functional implications that can further inform the development of novel biologic therapies. Here, we report the first quantitative phosphoproteomic analysis of a neuroblastoma cell line. This analysis implicated several intracellular signaling pathways that may be essential to neuroblastoma biology. In addition, we identified a number of phosphopeptides from protein tyrosine kinases that are activators of these pathways and potential oncogenic drivers of neuroblastoma cell growth, including IGF-1R/IR and Ret.

Our analysis identified 2181 non-redundant phosphorylation sites from 1171 unique proteins. The phosphoproteome of the *MYCN*-amplified tumor cells was characterized by high levels of phosphopeptides, including numerous mediators of intracellular signaling cascades. We identified an overabundance of phosphopeptides from protein kinases in these neuroblastoma cells of which a significant proportion are tyrosine-specific. Importantly, protein kinases constitute less than 2% of all human proteins, of which only 17% are tyrosine-specific [93]. Phosphopeptides from several RTK’s that are known for their oncogenic roles in other human tumors were identified in this neuroblastoma cell line, including IGF-1R/IR, FGFR1, FGFR2, Ret, and DDR2. Phosphopeptides from non-receptor tyrosine kinases were also observed at high levels included mTor, Raf, Chk1, CDK1, CDK2, GSK3β, JAK2, ABL2, and Lyn.

 IGF-1R signaling has been previously implicated in neuroblastoma biology, as well as in other human cancers [94-96]. IGF-1 has been described as affecting neuroblastoma growth and metastasis, including actin cytoskeletal rearrangement, enhanced cell motility, and neurite outgrowth [94,97]. IGF-1R signaling is primarily mediated through the IRS-1 and IRS-2 substrates, which have distinct functions [64,98,99]. Specifically, IRS-2, not IRS-1, protects neuroblastoma cells from caspase-mediated apoptosis and promotes tumorigenesis [100]. As such, IGF-1R targeted monoclonal antibodies are currently being trialed in the clinical setting for patients with high-risk neuroblastoma. In addition, IGF-1R antibodies and small molecule inhibitors have been investigated as therapy for other malignancies, including breast cancer, colorectal cancer, non-small cell lung cancer, ovarian cancer, pancreatic cancer, prostate cancer, leukemia, and rhabdomyosarcoma [101]. Our results demonstrate high levels of phosphopeptides from IGF-1R/IR, as well as its major substrates, IRS-2 and Shc, in *MYCN*-amplified neuroblastoma cells. Our analysis therefore supports the importance of IGF-1R/IR signaling in neuroblastoma tumor biology.

Our study further implicates phosphorylated Ret and FGFR in their potential role as drivers of neuroblastoma tumorigenesis. Ret is a proto-oncogene that encodes an RTK that is highly expressed on neurons and has been extensively investigated in adult thyroid cancer [69]. Ret gain-of-function mutations are associated with medullary thyroid cancer and are the cause of the inherited cancer syndromes MEN 2A and 2B [102]. Ret has been shown to be highly expressed in neuroblastoma cells [103-107]. Mice overexpressing Ret develop tumors resembling neuroblastoma [108]. Ret expression has also been shown to increase in neuroblastoma cell metastasis [109]. Treatment with Vandetanib significantly inhibits cell viability in multiple neuroblastoma cell lines and xenograft models, as shown in our data and other studies [78,79]. Despite these findings, the specific role of Ret signaling in neuroblastoma remains unclear. Some reports have shown that Ret is required for retinoic acid-induced neuroblastoma cell differentiation [104,110]. Yet, the combination of Vandetanib and RA is more effective than Vandetanib alone in inhibiting neuroblastoma cell growth [78]. Here, we demonstrated elevation of phosphopeptides from Ret and its Shc3 substrate in a *MYCN*-amplified neuroblastoma cell line, suggesting activation of this pathway in these cells. Elevated levels of phosphopeptides from FGFR1 and FGFR2 were also observed at high levels in these neuroblastoma cells and are implicated in the tumorigenesis of many human cancers, including neuroblastoma [111,112].

The major downstream targets of both IGF-1R/IR and Ret are the PI3K/Akt/mTor and Raf/MEK/ERK intracellular signaling pathways [69,95]. FGFR1 and FGFR2 are also capable of activating these pathways [111]. These pathways control multiple diverse cellular functions including metabolism, cell cycle regulation, and cell growth as well as cell survival and apoptosis [113]. These pathways have been demonstrated to play a key role in tumor biology and are known to be dysregulated in many human cancers [114,115]. Here, we identified high levels of phosphopeptides from downstream mediators of these pathways, including PI3K p85β, PDK1, GSK3β, MEK2, ERK1, and ERK2, in *MYCN*-amplified neuroblastoma cells. Phosphopeptides from c-MYC, one of the major downstream targets of these pathways, was also highly abundant in the neuroblastoma cells. Furthermore, we specifically demonstrated elevated levels of phosphopeptides from the oncogenic kinase mTor, including its cofactors PRAS40 and raptor, as well as its downstream targets p70S6K and 4E-BP1 in neuroblastoma cells. Inhibitory phosphorylation was also demonstrated on the tumor suppressor FOXO3 and pro-apoptotic protein BAD in our analysis. The DNA damage response pathway is also identified in our analysis, with high levels of phosphopeptides from DDR2 and Chk1. Taken together, our work reveals high levels of IGF-1R/IR and Ret phosphopeptides, with associated high abundance of phosphopeptides from downstream mediators of the PI3K/Akt/mTor and Raf/MEK/ERK pathways in *MYCN*-amplified neuroblastoma. 

Monoclonal antibodies and small molecule inhibitors are now frequently incorporated in clinical trials for the treatment of many human cancers. The identification of appropriate molecular targets in specific malignancies is challenging and requires an advanced understanding of the intracellular signaling pathways driving tumorigenesis. Our study demonstrates the ability of phosphoproteomics to identify both individual peptides and the specific post-translational modifications of those peptides within the global framework of a biological system. This approach should more readily allow for identification of novel therapeutic targets. Specifically, our work suggests that combined inhibition of both the PI3K/Akt/mTor and Raf/MEK/ERK pathways may significantly alter neuroblastoma cell viability. It also supports the targeting of IGF-1R, Ret and Chk-1, either alone or in combination.

Several genome-wide studies of neuroblastoma have been performed using next generation sequencing technology [116-119]. These studies report a low frequency of recurrent somatic mutations in these heterogeneous tumors. Somatic mutations in *ALK* have been reported in 7% of neuroblastoma cases, with mutations in *TIAM1* reported in 3% [117]. Phosphopeptide products from these genes were not identified in our proteomic analysis. Chromosomal deletions and sequence alterations in chromatin remodeling genes *ARID1A* and *ARID1B* were recently identified in 11% of neuroblastoma cases using massively parallel sequencing techniques [119]. Interestingly, our analysis found significant overabundance of multiple phosphopeptides of ARID1A in the NB10 cell line. Genetic alterations in regulators of the Rac/Rho pathway have also been found to occur in neuroblastoma tumors, which act to control neurogenesis [117]. Multiple phosphopeptides of Rac/Rho pathway mediators were noted as overexpressed in NB10 cells in our analysis as well, including Trio and ARHGAP17. In addition, multiple phosphopeptides of ATRX were identified as highly abundant in our analysis. Recently, loss-of-function mutations in this chromatin-remodeling gene have been described as a recurrent finding in neuroblastoma samples [116,118]. 

## Conclusions

In this work we demonstrate that *MYCN*-amplified neuroblastoma cells contain elevated levels of multiple phosphopeptides from receptor tyrosine kinases capable of activating several downstream signaling pathways. These complex signaling cascades are able to interact with each other to drive cell growth, enhance proliferation, and inhibit apoptosis. As such, targeting individual receptors or single downstream mediators within these pathways may not provide the most effective therapeutic approach. Our analysis, although limited in scope to a single *MYCN*-amplified cell line, identified many elevated phosphopeptides from proteins within the neuroblastoma phosphoproteome that could be targeted therapeutically to inhibit tumor growth. These results support an approach in which multiple receptors and downstream mediators should be targeted in combination in order to achieve maximum inhibition of neuroblastoma cell growth and therefore improved therapeutic gain. Isolated targeting of IGF-1R/IR, Ret, PI3K, MEK, and mTor has been trialed in neuroblastoma, with or without traditional chemotherapy [78,79,120-125]. We propose targeting these RTK’s and downstream signaling proteins in combination in order to inhibit both the PI3K/Akt/mTor and Raf/MEK/ERK pathways simultaneously. Our work demonstrates the power of a phosphoproteomic approach in the investigation of signaling pathways and potential identification of novel therapeutic targets.

## Supporting Information

Methods S1 **T statistics for comparison between NPC cell line and NB10 cell line for each phosphopeptide.**
(DOCX)

Table S1 **Identified phosphopeptides from both the TiO and IAP analyses.** Phosphorylation sites of all identified phosphopeptides with their corresponding protein name and gene symbols are listed. The ratio was calculated as the log {NB10 SIC peak area ÷ NPC SIC peak area}. Values greater than 0 indicate a relative increase in phosphopeptide abundance in the NB10 cells, while values less than 0 indicate a relative increase in phosphopeptide abundance in the NPC cells.
(XLSX)

Table S2 **Identified protein kinase phosphopeptides in the NB10 and NPC cell lines.** Phosphorylation sites of all identified protein kinase phosphopeptides with their corresponding protein names and gene symbols. The ratio was calculated as the log {NB10 SIC peak area ÷ NPC SIC peak area}. Values greater than 0 indicate a relative increase in phosphopeptide abundance for each individual phosphopeptide in the NB10 cells, while values less than 0 indicate a relative increase in the NPC cells.
(XLSX)

Table S3 **Highly abundant tyrosine phosphopeptides in NB10 relative to NPC cells.** Tyrosine phosphopeptides with the greatest differential in abundance in NB10 relative to NPC as determined from the IAP analysis. The ratio was calculated as the log {NB10 SIC peak area ÷ NPC SIC peak area}.
(XLSX)

Dataset S1 **Quantitative and statistical analysis of all identified phosphopeptides from the TiO protocol.** Sequence and phosphorylation site assignments of all identified phosphopeptides with their corresponding SIC peak areas and statistics (standard deviation, p-values and q-values) in both the NPC and NB10 cell lines.
(XLS)

Dataset S2 **Quantitative and statistical analysis of all identified tyrosine phosphopeptides from the IAP protocol.** Sequence and phosphorylation site assignments of all identified phosphopeptides with their corresponding SIC peak areas and statistics (standard deviation, p-values and q-values) in both the NPC and NB10 cell lines.
(XLS)
