# Supplementary material for: Quantitative Phosphoproteomic Analysis Identifies Activation of the RET and IGF-1R/IR Signaling Pathways in Neuroblastoma
Source: PLoS One. 2013 Dec 11;8(12):e82513. doi: 10.1371/journal.pone.0082513 (PMC3859635; doi:10.1371/journal.pone.0082513)
Supplement: Methods S1 — T statistics for comparison between NPC cell line and NB10 cell line for each phosphopeptide. (DOCX) [file pone.0082513.s001.docx]

**Supplemental Methods S1.**

T statistics for small data set:

T statistics for comparison between NPC cell line and NB10 cell line for each phosphopeptide were calculated according to the following equation:

$$T=\frac{(\bar{X_{1}}-\bar{X_{2}})}{S_{p}\sqrt{\frac{1}{n_{1}}+\frac{1}{n_{2}}}}$$

$$S_{p}=\frac{\left( n_{1}-1 \right)S_{1}^{2}+\left( n_{2}-1 \right)S_{2}^{2}}{n_{1}+n_{2}-2}$$

$$df=n_{1}+n_{2}-2$$

$n_{1},n_{2}:$ number of replicates

$S_{1}^{2},S_{2}^{2}:$ sample variance

$\bar{X_{1}},\bar{X_{2}}:$ average of peak area

$df$: degree of freedom
